# Development of a Long Period Fiber Grating Interrogation System Using A Multimode Laser Diode

**DOI:** 10.3390/s21030749

**Published:** 2021-01-22

**Authors:** Luís Henrique Silva, Paulo Santos, Luís C. C. Coelho, Pedro Jorge, José Manuel Baptista

**Affiliations:** 1 Faculty of Exact Sciences and Engineering, University of Madeira, Campus da Penteada, 9020-105 Funchal, Portugal; 2008716@student.uma.pt; 2 Department of Physics and Astronomy, Faculty of Sciences, University of Porto, R. Campo Alegre 687, 4169-007 Porto, Portugal; paulo.s.santos@inesctec.pt (P.S.); pedro.jorge@fc.up.pt (P.J.); 3 INESC TEC—Center of Applied Photonics, R. Campo Alegre 687, 4169-007 Porto, Portugal; luis.c.coelho@inesctec.pt

**Keywords:** interrogation technique, laser tunability, long period fiber grating, multimode laser, optical fiber sensor

## Abstract

Optical fiber gratings have long shown their sensing capabilities. One of the main challenges, however, is the interrogation method applied, since typical systems tend to use broadband light sources with optical spectrum analyzers, laser scanning units or CCD (Charged Coupled Device) spectrometers. The following paper presents the development of an interrogation system, which explores the temperature response of a multimode laser diode, in order to interrogate long period fiber gratings. By performing a spectral sweep along one of its rejection bands, a discrete attenuation spectrum is created. Through a curve fitting technique, the original spectrum is restored. The built unit, while presenting a substantially reduced cost compared with typical interrogation systems, is capable of interrogating along a 10 nm window with measurement errors reaching minimum values as low as 0.4 nm, regarding the grating central wavelength, and 0.4 dB for its attenuation. Given its low cost and reduced dimensions, the developed system shows potential for slow-changing field applications.

## 1. Introduction

Optical fiber sensors have been in development for about 50 years, and have since gained the attention of different industries, such as chemical, medical, and even telecommunications. This interest is motivated by their typically small size, dielectric nature (providing immunity to EMI - Electromagnetic Interference), and high sensitivity and resolution [1].

In particular, long period fiber gratings (LPFG) excel on these characteristics, making them an interesting choice for various sensing systems.

Long period fiber gratings are created by imposing periodic variations on a fiber’s refractive index, allowing the coupling of fundamental core mode to forward propagating cladding modes. The following equation, commonly known as the phase-matching condition, describes this behavior [2]:

(1) $$\lambda_{res}=\left(n_{01}^{co}-n_{m}^{cl}\right)\Lambda$$

*λ_res_* represents the resonant wavelength, which will couple to a cladding mode, $n_{01}^{co}$ is the fundamental core mode effective refractive index, $n_{m}^{cl}$ is the effective refractive index of the *m_th_* cladding mode, to which the core mode will couple, and *Λ* is the grating period.

External parameters such as temperature, strain, and external refractive index serve to change the phase-matching condition parameters, giving rise to changes on the coupled wavelengths. These sensitivities are thoroughly described in [3] and are the basis for using these elements as sensing devices. Moreover, such sensitivities can be enhanced by submitting the grating to different procedures. As an example, Du et al. [4] presented an etching technique, allowing a higher sensitivity regarding temperature and refractive index.

Besides the main parameters described above, the LPFG technology has matured to a point that allows more complex measurands, such as biomass and chemical detection [5], torsion [6] and even air quality [7].

One of the challenges of real-world implementation of these devices as sensors is the interrogation technique applied. Typical laboratory applications use broadband light sources and optical spectrum analyzers, laser scanning units, or CCD (Charged Coupled Device) spectrometers. These configurations may reach over thousands of euros, and such cost makes them impossible to implement in all real-world scenarios.

A few interrogation techniques have been proposed, presenting themselves as less expensive solutions.

In [3], a technique is proposed in which wavelength variations translate to intensity modulation. This is made by tuning a narrow spectral linewidth LED, or laser diode, on the high slope zone of a long period fiber grating rejection band. When the central wavelength changes, the attenuation applied to the light coming from the LED (or laser) will change as well. With a system calibration, one could detect such changes with a photodiode. This method is considerably cheaper when compared with traditional systems.

In [8], the method described is based on an arrayed-waveguide grating (AWG), with each end connected to a photodiode. The grating is illuminated through a broadband light source and connected to the AWG. This configuration allows the creation of a discrete spectrum, and with a curve fitting technique, the attenuation band is recreated.

Another interrogation technique is proposed in [9], where three distributed feedback lasers are used. These lasers were centered along with the 1530–1570 nm window, and each allowed to be thermally tuned along a 4 nm range. The purpose was to measure three 4 nm segments of one attenuation band of the grating, and then, resorting to a curve fitting algorithm, the attenuation band could be restored.

Ferreira et al. [10] presented a demodulation technique for a Fiber Bragg Grating using a low-cost multimode laser diode. The operating principle relies on current dithering, modulating the laser’s wavelength, and sweeping along the reflection band of the Bragg Grating while measuring the reflected light.

Furthermore, advancements on portable devices and, more specifically, smartphones, present a promising future regarding these devices capabilities as handheld spectroscopy devices. In [11], a smartphone camera adapter is presented, enabling this device to be used as a spectrum analyzer.

## 2. Materials and Methods

**Figure 1:**
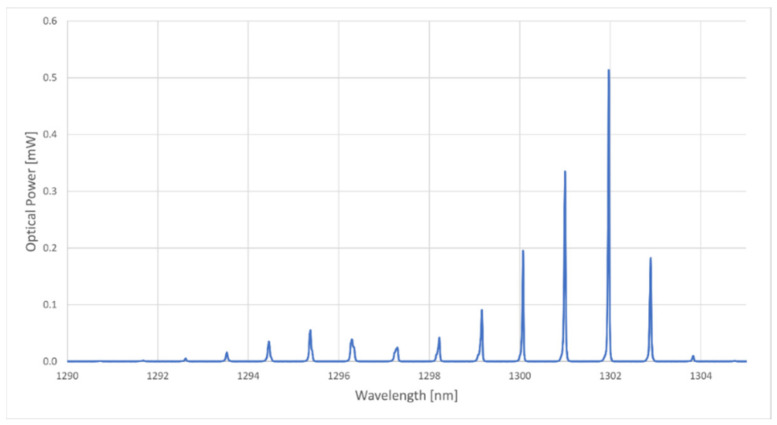
Optical spectrum of the employed laser at the ambient temperature of 23 °C and a constant current of 25 mA.

The operating principle of the developed unit was based on the temperature response of a Fabry–Pérot cavity laser diode. According to the manufacturer of the laser, QPhotonics, model QFLD-1310-5SAX (QPhotonics, Ann Arbor, MI, USA) (whose spectrum can be seen in Figure 1), had a temperature coefficient of 0.5 nm/°C. All spectra measurements, excluding the ones from the built interrogation unit, were performed with a Yokogawa AQ6370 optical spectrum analyzer.

Given its temperature range, between 0–60 °C, one could achieve a theoretical spectral sweep of 30 nm.

**Figure 2:**
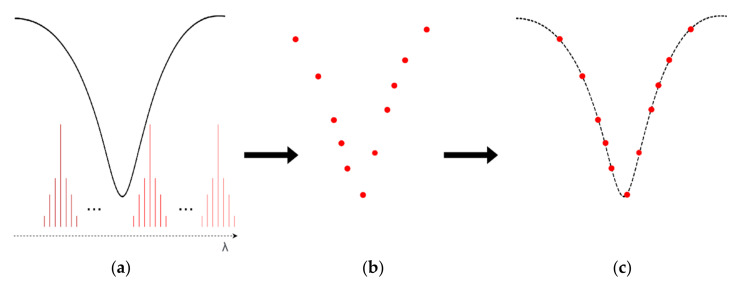
Operating principle of the developed interrogation system. (**a**): Spectral sweep by thermally tuning the laser’s wavelength; (**b**): Discrete spectrum resulting from the interrogation; (**c**): Reconstruction of the original spectrum by a curve fitting technique.

The following figure depicted the operating principle of the developed interrogation system. Figure 2a shows the thermal tuning of the laser’s wavelength, allowing for sweeping the spectrum and measuring the curve attenuation of the LPFG resonance. This was made by stabilizing the laser at discrete temperatures and comparing the emitted laser power with the power received on the other side of the grating. The successive measurements were made along the spectrum, creating the profile shown in Figure 2b. By resorting to a Gaussian curve fitting technique, the original attenuation spectrum was recreated, as depicted in Figure 2c.

**Figure 3:**
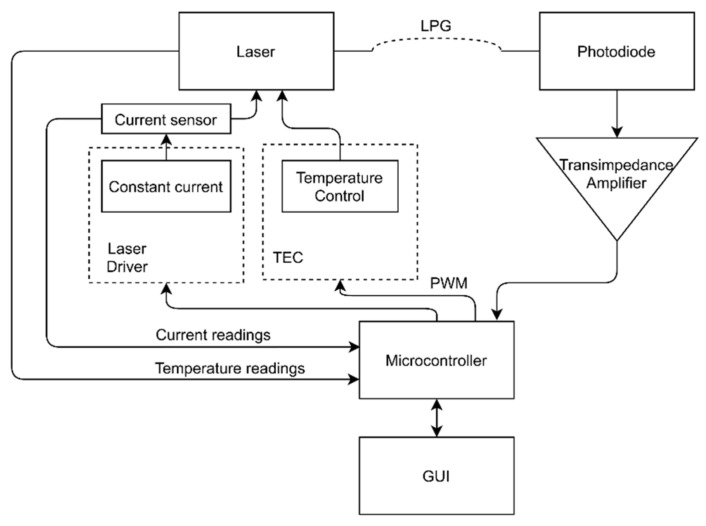
Block diagram of the developed interrogation system.

The portable system created consisted of a circuit board, a laser diode, a laser enclosure (allowing temperature monitoring and control), a photodiode (model PF512), and a microcontroller. The whole system was powered by a 7.5 V DC power supply. The system’s block diagram is shown in Figure 3.

The developed electronic circuit feeds the laser a constant limited current while also supplying electrical power to the other parts of the circuit, such as the temperature control system, optical power measurements, and the laser temperature and current reading circuits. The last being accomplished by a current sensor installed in series with the laser.

In order to power the laser, a low cost and commercially available laser driver integrated circuit (MAX3668) (Maxim Integrated, San Jose, CA, USA) was used, feeding a constant current of 25 mA to the laser. The driver acquired allowed currents up to 80 mA, giving a degree of flexibility to the system, enabling the use of more powerful laser diodes.

Optical power readings on the exit side of the grating were done by a photodiode connected to a variable gain transimpedance amplifier. The transimpedance amplifier converted optical power values to voltage values. The gain was adjusted thus that the voltage output values did not go above the maximum allowed by the microcontroller, in this particular case being 3.3 V.

The system control and voltage readings were carried out by the microcontroller, which periodically communicated with a graphical user interface, GUI, created in LabVIEW. The communication was done via a UART protocol.

**Figure 4:**
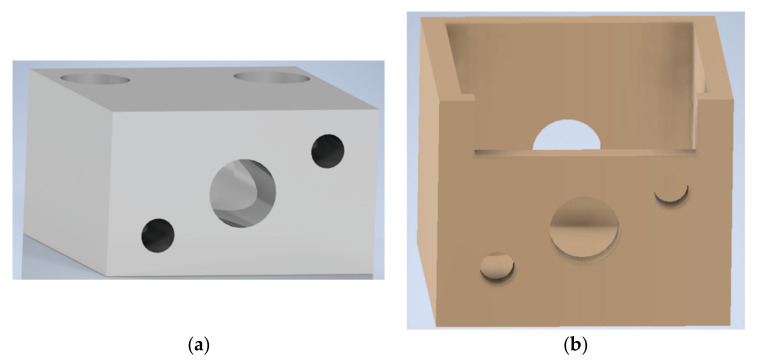

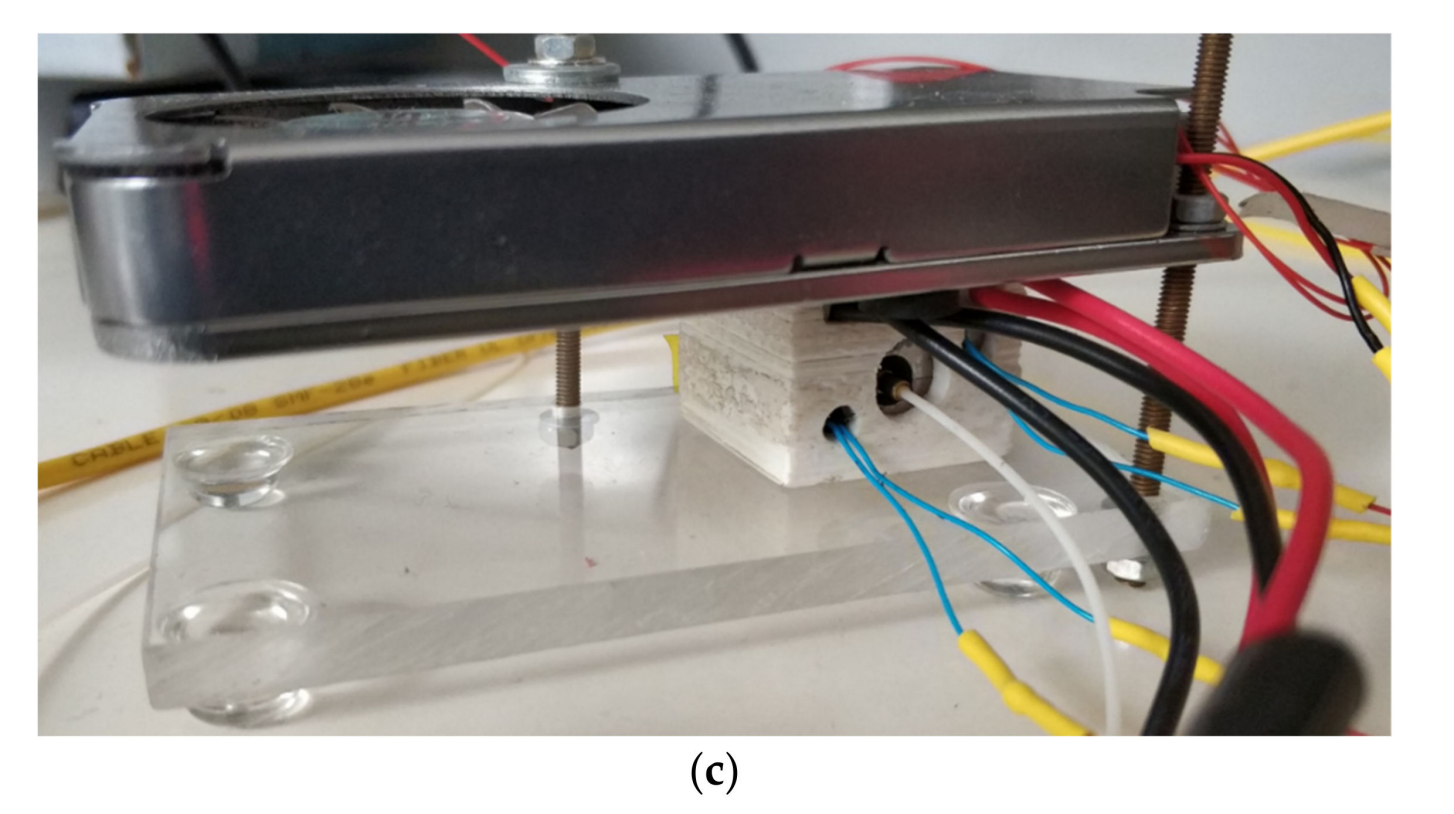
Images of: (**a**) Rendered aluminum block created to accommodate the laser diode and two thermistors; (**b**) rendered 3D printed shell to accommodate the aluminum block and contain possible condensations; (**c**) developed laser holding and enclosure structure.

To change the laser temperature, an enclosure aluminum block was created by a CNC machine, shown below in Figure 4a.

The laser was installed inside the block using a thermal paste to enhance the heat transfer between the 2 bodies. Two holes were made on one side of the block, allowing the installation of 2 thermistors, providing real-time temperature measurements. These measurements were carried out by the microcontroller and were shown on the graphical user interface while also serving as a reading to a temperature control algorithm.

In addition, to contain any possible condensation originating from cooling the block to temperatures below ambient temperature, a 3D shell was printed to fit the aluminum enclosure. The design is depicted in Figure 4b.

The heating and cooling elements used were 2 thermoelectric coolers (TEC) installed on the top of the aluminum block. Once again, the thermal paste was used in order to provide better thermal conductivity. To control the block’s temperature, a pulse width modulation (PWM) signal was used on the TEC supply. The duty cycle was calculated through a proportional controller, attributing a gain to the temperature error, measured between the reference temperature and the temperature read by the thermistors. In order to limit the current consumption from the TEC elements, a maximum duty cycle was imposed via software.

Since typical TEC elements require heat dissipation when cooling, a heatsink and fan were installed on top of the TEC elements, allowing the system to reach temperatures under ambient temperature. The assembly of all these components can be seen on Figure 4c.

The total cost of the materials used was below €380, with the laser diode being the most expensive component with a cost of about €160. This implementation was significantly cheaper when compared with typical configurations that used broadband light sources with optical spectrum analyzers, laser scanning units, or CCD spectrometers.

The grating used in this experiment was fabricated in a standard single mode fiber SMF28, using the induced electric arc technique, with a modulation period of 320 μm, presenting an attenuation band around 1322 nm [12].

## 3. Results

### 3.1. Laser Temperature Characterization

**Figure 5:**
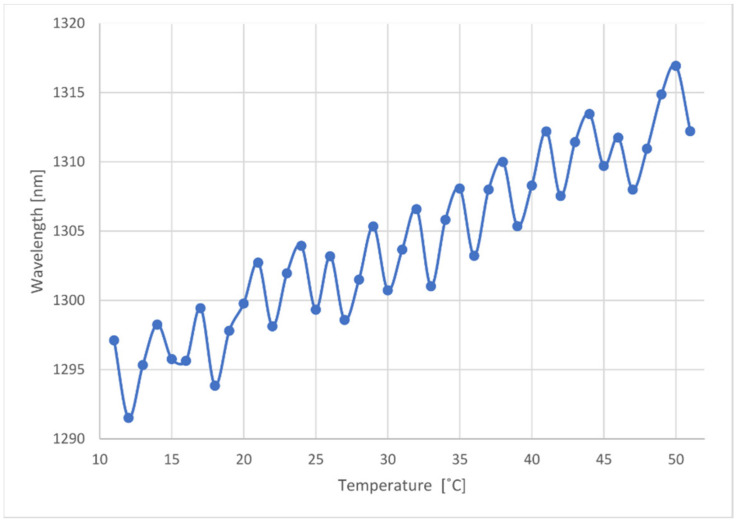
The wavelength of the laser’s dominant mode as a function of temperature, for a constant current of 25 mA.

To better understand the laser’s wavelength behavior with varying temperatures, a characterization was made. For this, a constant current of 25 mA was supplied, and the laser temperature was increased between 11 °C and 51 °C, with steps of 1 °C. As a precautionary measure, the full temperature range allowed by the laser was not used. In addition, for each temperature, a few minutes were given to ensure the laser had reached thermal stability. The characterization was performed considering the wavelength of the mode with the highest power for each temperature. It should be noted that, depending on the temperature, the dominant mode varied, resulting in the behavior presented below. Figure 5 shows the wavelength change of the dominant mode as a function of temperature.

**Figure 6:**
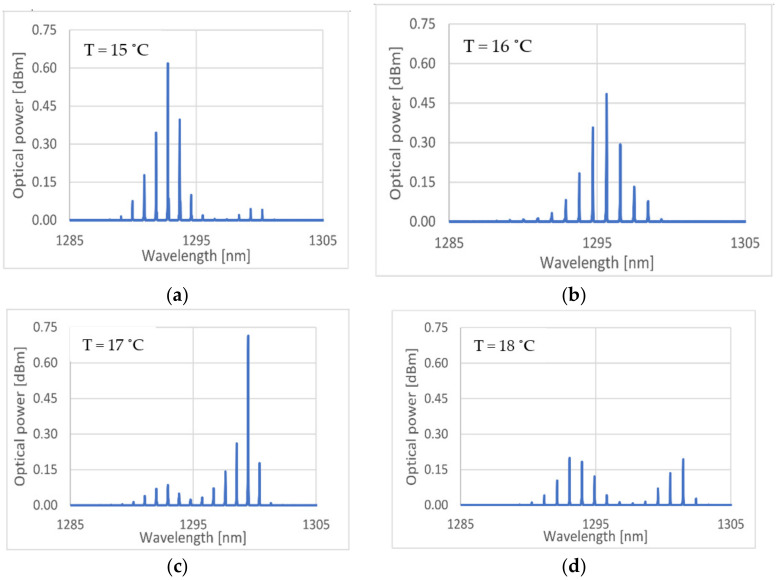
Spectral profiles presented by the laser, depending on the operating temperature, for a constant current of 25 mA. (**a**) Laser spectrum for a temperature of 15 °C; (**b**) laser spectrum for a temperature of 16 °C; (**c**) laser spectrum for a temperature of 17 °C; (**d**) laser spectrum for a temperature of 18 °C.

It can be seen that the wavelength drifts to higher values as temperature increases, as expected. The periodic oscillation occurred due to the periodic variations of the dominant mode of the laser. Figure 6 shows typical spectral profiles when the laser was biased with a constant current of 25 mA, and for different temperatures. As can be observed, for different temperatures, energy was transferred between different modes in a determined pattern. These profiles tended to repeat as temperature changes. Therefore, it was possible to have a stable and precise measuring system. On the other hand, the high sampling numbers measured will help to mitigate any eventual fluctuations in the stability and precision of the measuring system.

The wavelength with temperature characterization was done considering the fundamental mode, since in the spectra considered (Figure 6a–c)) most of the optical power was distributed between the fundamental mode and the two adjacent neighboring modes, being these three modes the most relevant in terms of amplitude contribution for the mode integration at the photodetector.

Considering the behaviors both shown on Figure 5 and Figure 6, the laser characterization was made in a discrete manner. The chosen temperatures, for which the laser will be stabilized during the interrogation, were all that presented a stable, and not too broad spectral profile. A direct correspondence between the temperature and the laser’s dominant mode wavelength was made on the microcontroller.

During a wavelength sweep, the system stabilized the laser temperature on the various chosen temperatures, successively. For each one, the grating attenuation was measured by comparing the laser output power with the power received by the photodiode on the exit end of the grating. Multiple optical power measurements were performed for each measured point. This, in turn, allowed a time averaging in order to compensate any power fluctuations that might occur on the optical source.

**Figure 7:**
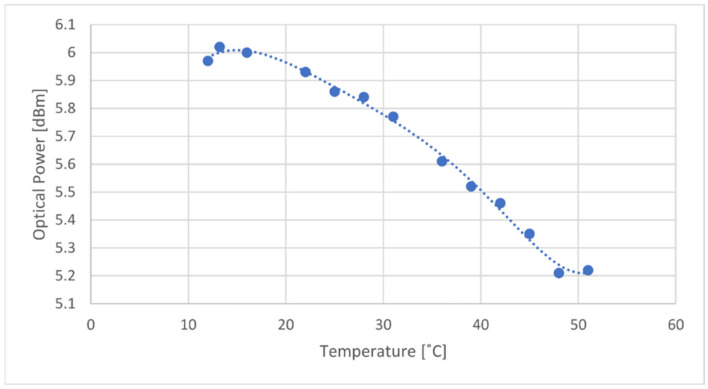
Laser emitted power as a function of temperature for a constant current of 25 mA.

Since the laser was operated with a constant bias current of 25 mA, another characterization between the laser’s temperature and the output power was done. For this, the laser was maintained with a constant current of 25 mA, and the output power was measured for 13 values along with the temperature range. The relation can be seen in Figure 7.

Optical power varies about 0.8 dBm between both ends of the temperature range, decreasing as temperature increases, which is coherent with the literature [13]. In order to compensate for this variation, a polynomial curve, of degree 6, was shown to be the best fit for the measured values. This equation will allow the system to predict the optical power emitted from the laser, given its operating temperature.

Some sort of optical power degradation was expected as a result of system aging [13]. Such degradation was normally difficult to predict, and as it happens with all types of measuring systems, thus the proposed interrogation system should be periodically calibrated.

### 3.2. Photodiode Characterization

The photodiode used, model PF512, was connected to an adjustable gain transimpedance amplifier circuit, converting optical power to voltage values, readable by the microcontroller’s ADC (analog-to-digital converter).

**Figure 8:**
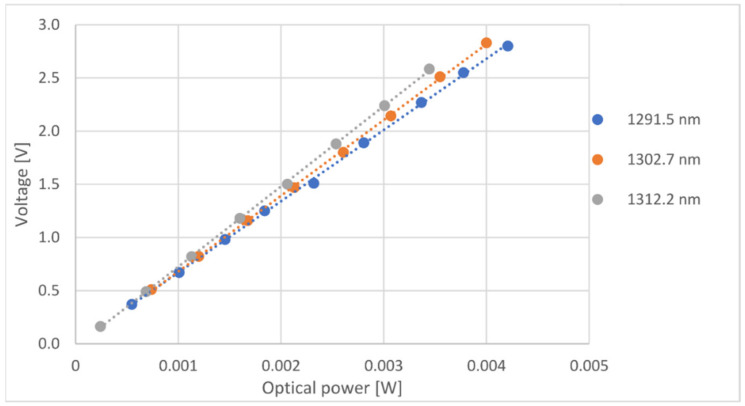
Relationship between the optical power received by the photodiode and the voltage at the transimpedance amplifier output for three distinct wavelengths.

The characterization was done by thermally tuning the laser on different wavelengths. For each wavelength, optical power changes were made by varying the supplied current. Each optical power change was recorded, along with the voltage presented by the transimpedance amplifier output. Figure 8 shows the relation between the optical power received by the photodiode and voltage output, for three distinct wavelengths.

Table 1 presents the linear equations and R^2^ values for all three wavelengths:

The voltage output as a function of the received optical power shows very high linearity. It can also be seen that the slope tends to increase as the wavelength gets higher, meaning a higher responsivity for higher wavelengths. These results were expected and were coherent with the photodiode’s datasheet.

Calculating the slope of the output voltage over the input optical power for every wavelength used, another graph was created for the slope variation as a function of wavelength. Such graph can be seen below.

**Figure 9:**
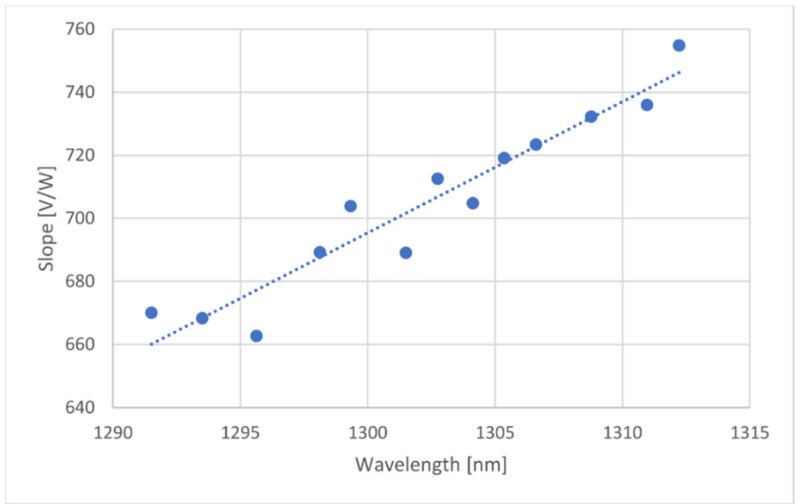
Input optical power over the output voltage slope as a function of the wavelength.

A linear regression was applied over the data (seen on Figure 9), and the equation was programmed on the microcontroller, allowing compensation for the varying responsivity as a function of the wavelength change.

### 3.3. Experimental Results

The evaluation of the interrogation system’s performance was done using a long period fiber grating. The wavelength of the attenuation peak was tuned by employing different and arbitrary, curvature radius on the grating, allowing multiple interrogations along the measurable spectrum, with high stability along the measurement time.

**Figure 10:**
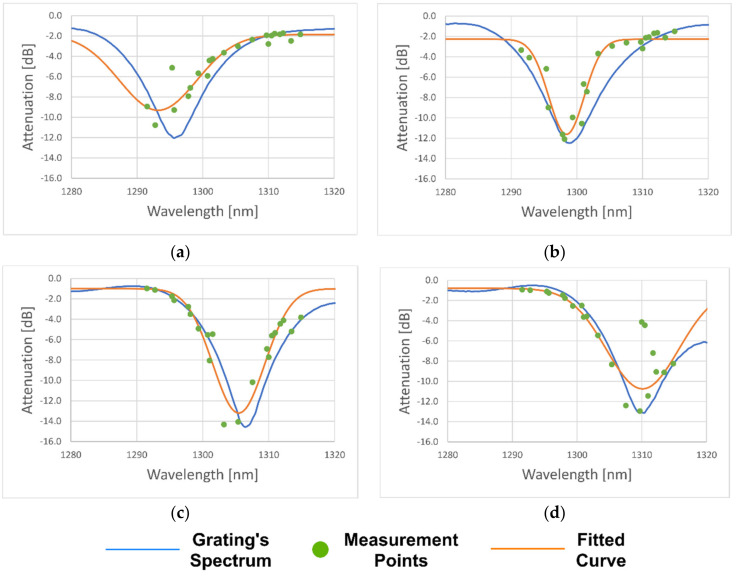
Interrogations done along the measurable spectrum, the blue line represents the grating spectrum measured with an optical spectrum analyzer, green dots are the measurement points, and the orange line represents the fitted Gaussian curve. (**a**) long period fiber gratings (LPFG) centered at 1295.6 nm; (**b**) LPFG centered at 1298.8 nm; (**c**) LPFG centered at 1306.4 nm; (**d**) LPFG centered at 1310.0 nm.

Figure 10 shows the results obtained from four different interrogations.

Table 2 compares and summarizes the results obtained.

Compared to the results obtained from the built interrogation unit, Figure 10a presents a significantly higher error regarding wavelength, which can be attributed to the fact that not enough points were measured on the lower side of the spectrum. In addition, a few outliers can be seen, which are a factor that contributes to increasing the error.

In the particular case of Figure 10d, the small wavelength error was attributed to the highly accurate profiling of the left tail of the grating spectrum, contributing to a very approximate curve fitting. The outliers observed on this interrogation presented a larger influence on the amplitude error rather than the central wavelength.

The outliers are caused by the fact that the laser used was multimode, occasionally giving rise to power transfer between modes and having a reasonably large spectrum. Since the photodiode received a convolution of the laser and the grating spectra, the calculated attenuation may not be exactly the same as the real attenuation on the considered wavelength.

However, it was observed that when the central wavelength of the grating was close to the edges of the interrogation range, the error tended to increase considerably. As said above, this was caused by the fact that the system measured very few points on one tail of the grating spectrum. This situation, allied with the presence of outliers, reduced the interrogation window, where the results tended to be consistent, and the errors were significantly lower.

Based on the results achieved, the measurable spectrum range lay between 1298–1308 nm, presenting minimum error values of 0.4 nm and 0.4 dB, regarding the central wavelength and peak attenuation, respectively. Gratings centered between this range were guaranteed to have enough measured points on both sides, ensuring a good interrogation.

It is important to point out that during an interrogation cycle, when the temperature reached the target value, the system waits two minutes to ensure thermal stabilization of the laser and then performs the optical power measurements. Given this principle of operation that relies on temperature modulation and stabilization, the system has a slow response time, taking approximately 50 min to complete a full interrogation cycle.

Therefore, the interrogation system was intended for slow-changing applications, such as monitoring slow chemical reactions, for instance, corrosion, long-term environmental monitoring, as an example, the monitoring of saline intrusion in coastal aquifers, or slow cooling or slow heating processes like materials’ curing. Such processes are potential application candidates.

## 4. Conclusions

In this paper, a long period fiber grating interrogation system was proposed. The operating principle is based on the thermal coefficient of a multimode Fabry–Pérot laser diode. By thermal tuning the laser on different wavelengths, it was possible to measure the grating attenuation by comparing the emitted power versus the received power, creating a discrete attenuation spectrum. Using a Gaussian curve fitting technique, the original spectral profile was reconstructed.

The developed interrogation system presented an interrogation spectral window of about 10 nm, ranging from 1298 nm to 1308 nm, presenting errors as low as 0.4 nm and 0.4 dB, regarding the central wavelength and peak attenuation, respectively. The interrogation window can be increased by adding other lasers centered on adjacent wavelengths. Alternatively, one could add a laser centered at a completely different range, potentially enabling the system of multiple interrogations at the same time.

Although curvature was the measurand used to evaluate the system’s performance, it could be applied to different scenarios, such as temperature, strain, or refractive index measurements. As stated before, the interrogation system is intended for slow changing applications, such as monitoring slow chemical reactions, for instance, corrosion, long-term environmental monitoring, as an example, the monitoring of saline intrusion in coastal aquifers, or slow cooling or slow heating processes like materials’ curing.

In terms of the system’s total cost, around €380, it lies well under the typical interrogation systems using optical spectrum analyzers and broadband light sources, laser scanning units or CCD spectrometers, making it an affordable system, suitable for less demanding sensing applications.

## Figures and Tables

**Table 1:** Linear equations and R^2^ values for three wavelengths, with P the optical power in watts.

Wavelength [nm]	Equation	R^2^
1291.5	670.08×P−0.0004	0.9994
1302.7	712.64×P−0.0334	0.9996
1312.2	754.86×P−0.0300	0.9998

**Table 2:** Comparison of the broadband light source and OSA (Optical Spectrum Analyzer) results with the results obtained from the built interrogation unit.

OSA Central Wavelength [nm]	OSA Attenuation Peak [dB]	Measured Central Wavelength [nm]	Measured Attenuation Peak [dB]	Wavelength Central Error [nm]	Attenuation Peak Error [dB]
1295.6	−12.0	1293.2	−9.3	2.4	2.7
1298.8	−12.5	1298.4	−11.6	0.4	0.9
1306.4	−14.6	1305.5	−13.2	0.9	1.4
1310.0	−13.1	1310.1	−10.7	0.1	2.4

## Data Availability

Not applicable.
